# Action Mechanisms of Lithium Chloride on Cell Infection by Transmissible Gastroenteritis Coronavirus

**DOI:** 10.1371/journal.pone.0018669

**Published:** 2011-05-06

**Authors:** Xiaofeng Ren, Fandan Meng, Jiechao Yin, Guangxing Li, Xunliang Li, Chao Wang, Georg Herrler

**Affiliations:** 1 College of Veterinary Medicine, Northeast Agricultural University, Harbin, China; 2 College of Life Sciences, Northeast Agricultural University, Harbin, China; 3 Institut für Virologie, Tierärztliche Hochschule Hannover, Hannover, Germany; The Scripps Research Institute, United States of America

## Abstract

Transmissible gastroenteritis virus (TGEV) is a porcine coronavirus. Lithium chloride (LiCl) has been found to be effective against several DNA viruses, such as Herpes simplex virus and vaccinia virus. Recently, we and others have reported the inhibitory effect of LiCl on avian infectious bronchitis coronavirus (IBV) infection, an RNA virus. In the current study, the action mechanism of LiCl on cell infection by TGEV was investigated. Plaque assays and 3-(4,5)-dimethylthiahiazo(-z-y1)-3,5-di-phenyl tetrazoliumbromide (MTT) assays showed that the cell infection by TGEV was inhibited in a dose-dependent manner, when LiCl was added to virus-infected cells; the cell infection was not affected when either cells or viruses were pretreated with the drug. The inhibition of TGEV infection *in vitro* by LiCl was observed at different virus doses and with different cell lines. The inhibitory effect of LiCl against TGEV infection and transcription was confirmed by RT-PCR and real-time PCR targeting viral S and 3CL-protease genes. The time-of-addition effect of the drug on TGEV infection indicated that LiCl acted on the initial and late stage of TGEV infection. The production of virus was not detected at 36 h post-infection due to the drug treatment. Moreover, immunofluorescence (IF) and flow cytometry analyses based on staining of Annexin V and propidium iodide staining of nuclei indicated that early and late cell apoptosis induced by TGEV was inhibited efficiently. The ability of LiCl to inhibit apoptosis was investigated by IF analysis of caspase-3 expression. Our data indicate that LiCl inhibits TGEV infection by exerting an anti-apoptotic effect. The inhibitory effect of LiCl was also observed with porcine epidemic diarrhea coronavirus. Together with other reports concerning the inhibitory effect of lithium salts on IBV in cell culture, our results indicate that LiCl may be a potent agent against porcine and avian coronaviruses.

## Introduction

Transmissible gastroenteritis virus (TGEV) belongs to the family *Coronaviridae* and is one of the most important causative agents of enteric infections in pigs. The infection is associated with high morbidity in animals of all ages and with high mortality rates (up to 100%) in seronegative suckling piglets [1]-[3]. TGEV is an enveloped virus with a positive-stranded RNA genome approximately 28.5-kb in size, and it consists of four structural proteins: the spike (S), the integral membrane (M) glycoprotein, and the nucleocapsid (N) protein [1], [4], [5]. About two-thirds of the entire RNA from the 5′ end comprise open reading frames 1a and 1ab, which encode a number of nonstructural proteins including the replicase. The 3′ third of the genome contains the genes encoding the structural and some nonstructural proteins (5′-S-3a-3b-E-M-N-7-3′) [6]. The glycoprotein S is primarily responsible for inducing neutralizing antibodies and for initiating infection [7]-[9]. The appearance of porcine respiratory coronavirus (PRCoV), a respiratory mutant of TGEV has drastically decreased the risk of TGE in Europe, since neutralizing antibodies elicited by the avirulent PRCoV can provide cross-protection against TGEV infection [10]. In contrast, TGE prevalence is still reported and some TGEVs have been isolated in different parts of the world, e.g. in various geographical locations in China, implying that TGEV infection is still threatening pig industry [11]–[14].

At present, several commercially available vaccines are commonly used for prevention of TGEV infection in China. However, current traditional inactivated and attenuated vaccines are less effective than desired due to failure of vaccination to prevent viral shedding or reversion of the attenuated to a virulent phenotype. The lack of therapeutical treatment of TGE underlines the importance of development of effective antivirals Lithium salts have been used to treat diseases such as 'gout and rheumatic gout', 'Bright's disease', epilepsy, syphilis, acute mania and depressive episodes [15]. There are several reports regarding the inhibitory effect of lithium salts on the replication of several DNA viruses, such as type 1 and 2 herpes simplex virus and vaccinia virus [15], [16]. More recently, we and another research group demonstrated that lithium chloride (LiCl) inhibits infection of cell cultures by infectious bronchitis coronavirus (IBV), an avian coronavirus [17], [18].

The purpose of the current study was to investigate the action mechanism concerning the inhibitory effect of LiCl on cell infection by TGEV and to find out whether the susceptibility to LiCl treatment is also a feature of other RNA viruses. The effect of LiCl on TGEV infection was analyzed by plaque assays, RT-PCR and quantitative real-time PCR. The effect of the drug on infection cycle of TGEV and virus production was assessed by time-dependent drug addition. The inhibition of LiCl to cell apoptosis caused by TGEV was demonstrated by immunofluorescence and flow cytometry. The protective effect of LiCl to other RNA viruses was also compared. Our data demonstrate that LiCl may be a potent antiviral agent via an anti-apoptotic mechanism.

## Methods

### Cells and viruses

Swine testis (ST) cells and porcine kidney (PK-15) cells were maintained in Eagle's Minimum Essential Medium (EMEM). Monkey kidney cell lines (Vero) and (MA 104) cells were cultured in Dulbecco's Modified Eagle Medium (DMEM). All the cells were purchased from ATCC and kept in our laboratory. Cells were cultured in respective medium supplemented with 10% newborn bovine serum (NBS, Excell Bio. China) at 37°C in the CO_2_ incubator. TGEV strain PUR46-MAD was propagated in ST or PK-15 cells. Porcine epidemic diarrhea virus (PEDV) isolate HLJBY and Bovine rotavirus (BRV) strain NCDV were propagated in Vero cells and MA104 cells, respectively.

### Cytotoxic assay

Cytotoxic effect of lithium chloride, LiCl (Sigma, USA) on cell viability was analyzed by trypan blue staining as previously described [19]. Briefly, the cells in 6-well plates were incubated LiCl serially diluted in serum-free EMEM (6 wells/each dilution) for 48–72 h. After phosphate buffered saline (PBS) washing, the cells were incubated with 0.5% w/v Trypan Blue Mix diluted in PBS (1ml/well) at room temperature for 5 min followed by three times washing with PBS. The percentage of viable cells was calculated according to the equation: (Number of unstained cells/Total number of cells)×100 =  Percentage of viable cells. The effect of LiCl on cell proliferation was determined by 3– (4,5)-dimethylthiahiazo (-z-y1) −3,5-di-phenytetrazoliumbromide (MTT) assays. Briefly, cells in 96-well culture plates were washed three times with PBS and incubated with LiCl serially diluted in serum-free EMEM (6 wells/each dilution) for 48–72 h. Mock-treated cells served as control. After washing with PBS, the cells were incubated with medium (80 µl/well) and 0.5% MTT solution (20** µ**l/well) at 37°C for 4 h. The cells were incubated with dimethyl sulfoxide, DMSO (150** µ**l/well) and the tray was shaken gently for 10 min, after washing with PBS. Optical density (OD) value of the wells at a test wavelength of 570 nm was measured using an ELISA reader. Cell survival rate was determined as drug average OD value/control average OD value. The 50% cytostatic concentration (CC_50_) was defined as the concentration that inhibited the proliferation of exponentially growing cells by 50% and non cytotoxic concentration (≤CC50) of LiCl was used for antiviral assays.

### Virus titration and infection

For virus titration or infectivity analysis, plaque assays were performed as previously described with modifications [18], [20]. Briefly, cells seeded onto 24-well culture plates were inoculated with serially diluted viruses. The inoculums were then replaced with 1% (w/v) methylcellulose in EMEM (1 ml/well), after virus absorption at 37°C for 1 h. Forty-eight hours later, the overlaid medium was removed and the cells were rinsed three times with PBS. Then they were fixed with 3% formalin in PBS (200** µ**l/well) for 30 min at room temperature followed by staining with 1% crystal violet (w/v) diluted in 5% ethanol in PBS (200** µ**l/well) for 20 min. The plaque number was recorded after complete washing with PBS, and the virus titer in plaque-forming units (pfu) was calculated. All infection assays were performed in 24-well culture plates, unless otherwise indicated. Each assay was performed in triplicate.

### Antiviral activity of LiCl

To analyze the effect of LiCl on viruses, TGEV (1×10^6^ pfu/ml) was incubated with serially diluted LiCl in medium at 37°C for 1 h. Then the viruses at an MOI (multiplicity of infection) of 0.3, 3, or 30 were used to infect cells in 24-well plates at 37°C for 1 h. After washing three times with PBS, the cells were overlaid with 1% methylcellulose in medium and cultured for 48–72 h prior to plaque assays as above. To analyze the impact of LiCl on cell, cells in 24-well plates were treated with serially diluted LiCl in medium at 37°C for 1 h. The cells were infected with TGEV at an MOI of 0.3, 3, or 30, after the cells were washed with PBS for three times. Subsequently, the cells were overlaid with methycellulose for plaque assays as above. To investigate the effect of LiCl on TGEV-infected cells, cells in 24-well plates were washed with PBS for three times and then infected with TGEV at an MOI of 0.3, 3, or 30 at 37°C for 1 h. Thereafter, the cells were washed with PBS and incubated with serially diluted drugs at 37°C for 48–72 h. The infectivity of the viruses was calculated with plaque assays as above.

### Impact of LiCl on TGEV-infected cells

To analyze the effect of LiCl on proliferation of cells infected with TGEV, MTT colorimetric assays were performed [19], [21], [22]. Briefly, cells were plated in triplicate in 96-well plates. The cells were incubated with TGEV at an MOI of 0.3, 3, or 30 at 37°C for 1 h. After washing with PBS, the cells were incubated with LiCl at different concentrations at 37°C. Virus-infected and mock-treated cells were infection and blank controls, respectively. At 48 h post-infection (hpi), the cells were incubated with MTT solution (5 mg/ml in PBS, 10** µ**l/well) at 37°C for 4 h. After washing with PBS, the cells were incubated with DMSO and the OD_570_ value was read as described above. Relative amount of survival cells (%) was calculated as follows: Percent viable cells  = {[OD of drug treatment group-OD of infection control]/[OD of blank control-OD of infection control]}×100%.

### Reverse transcription-PCR

Reverse transcription (RT)-PCR was used to amplify (i) a partial S gene designated as S-AD (encoding the major antigenic sites A and D at the 5′ end half of the TGEV S gene), (ii) a partial gene comprising the 3′half of the S gene, the intervening sequence and the 3a gene (designated S-X), and (iii) a nonstructural gene encoding the 3CLprotease (3CLpro). The housekeeping gene, beta-actin was used as an internal reference. The information regarding primer sequences and amplification products are summarized in Table 1. TGEV-infected ST cells treated by LiCl in 6-well plates were subjected to RNA extraction with a commercial kit (Qiagen, Germany). Subsequent RT-PCR was performed using a RT-PCR kit (TaKaRa, Japan). The PCR profile for amplification of S-AD gene included 95°C for 10 min and 30 cycles of 94°C for l min, 56°C for 1 min, 72°C for l min. There was a final extension at 72°C for l0 min. The PCR for amplification of TGEV S-X gene, 3CL protease gene and beta-actin was performed as described for the S-AD gene except that the annealing temperature were 47.6°C, 53°C and 58.8°C, respectively. The PCR products were electrophoresed on 1% agarose gel.

**Table 1:** Information on primers and RT-PCR products.

Primer pairs	PCR product in length (bp)
Sense 5′-GGGCGAATTCATGACTCTTGAAATTTCATGTTAT-3′ Antisense 5′-CCGGGTCGACTTTTATAACAGCTGTGGCATCTAA-3′	TGEV S-AD (734)
Sense 5′-CTTAGTAGTAATATTTTGCATAC-3′ Antisense 5′-TATAGCAGATGATAGAATTAACA-3′	TGEV S-X (192)
Sense 5′-ATGAAGGATGTCCTGGCAGTGT-3′ Antisense 5′-ACCACCGTACATTTCTCCTTCAAA-3′	TGEV 3CLpro (200)
Sense 5′-GGCTCAGAGCAAGAGAGGTATCC-3′ Antisense 5′-GGTCTCAAACATGATCTGAGTCATCT-3′	Beta-actin (208)

### Real-time RT-PCR

The effect of LiCl on cell infection by TGEV was confirmed by real-time PCR targeting the S-X gene and 3CLpro gene of TGEV. ST cells in 6-well plates were infected with TGEV at an MOI of 30 at 37°C for 1 h. The cells were treated with LiCl at different concentrations at 37°C for 36–48 h, after washing with PBS. Then the virus-containing culture was frozen and thawed three times followed by addition of an equal volume of 20% polyethylene glycol (PGE) 8000 at room temperature for 30 min. The samples were centrifuged at 12000 rpm for 5 min and the pellets were resuspended in RNase-free water. Then the total RNA was extracted with a commercial kit (Fastgene, China) according to the manufacturer's instructions. The reverse transcription was performed in total of 20** µ**l volume consisting of cDNA 5** µ**l, oligo dT primer 1** µ**l, dNTP (10 mM) 1** µ**l, RNAse inhibitor 0.5** µ**l, sterile water 7.5** µ**l, MLV 1** µ**l, 5×RT-PCR buffer 4** µ**l. The reaction was performed at 30°C for 10 min, 42°C for 60 min, and 95°C for 5 min. Subsequent real-time PCR was performed using ABI PRISM 7500 real-time PCR machine (Applied Biosystems, USA). The real-time PCR included 0.5** µ**l cDNA template, 10** µ**l SYBR Taq polymerase, 0.4** µ**l ROX pge 2, 0.5** µ**l sense primer, 0.5** µ**l antisense primer and 8.1** µ**l sterile water. The PCR parameters included 95°C for 10 s; 40 cycles of 95°C for 5 s and 60°C for 34 s. The expression level of TGEV S-X and TGEV 3CLpro was normalized to that of beta-actin according to the comparative cycle threshold (*C_T_*) method used for quantification recommended by the manufacturer's protocol. Delta *C_T_* was measured. The expression of TGEV S-X and 3CLpro in TGEV-infected ST cells was normalized to that of beta-actin and taken as 100% compared to expression with drug treatments.

Data analysis is based on the measurement of the cycle threshold (Ct). Isolated total DNA from untreated sample was taken as reference. For each treated sample, the difference in △Ct (Ct sample fragment mean Ct value-beta-actin fragment mean Ct value) was used as a measure of the relative fragment with the 2^−△△Ct^ method [23], [24] in correlation to the amplification size of TGEV S-X or TGEV 3CLpro fragment. In brief, for the quantification of the respective fragment in the treated samples, real-time PCR analysis for the corresponding beta-actin fragments was performed. For each experimental condition, real-time PCR was conducted in quadruplicates and the resulting average of Ct values for the TGEV X-S or TGEV 3CLpro fragment was used for the evaluation of virus quantity. Therefore, the difference in the △Ct of untreated versus each treated condition of the respective TGEV X-S or TGEV 3CLpro fragment was calculated by the 2^−△△Ct^ method expressed as ratio of the reduction for both fragments. The experiment was performed in triplicate.

### Time course of drug addition and virus production

The time-of-addition effect of LiCl on TGEV infection was performed to analyze its mode of action. Cells in 96-well plates were infected with TGEV at an MOI of 30. The cells were incubated with LiCl at a concentration of 25 mM at 1 h intervals starting at 1 h post infection (hpi). The infectivity of the viruses was analyzed at the indicated time points using MTT assays by measuring OD_492_ value of each well as above. In parallel, to anazlye the effect of LiCl on virus production, the cells were infected with TGEV at an MOI of 30 for 1 h followed by incubation with serially diluted LiCl. Subsequently, the supernatants of the infected cells were collected at the indicated time points and subjected to plaque assays.

### Inhibition of other viruses

PEDV and BRV used to infect Vero and MA104 cells in 24-well plates at 37°C for 1 h. Then the culture was kept at 37°C for 36–48 h in the presence of LiCl at different concentrations. The infectivity of both viruses was determined by plaque assays. The assays were performed in triplicate.

### Cell apoptosis analysis

Cell apoptosis was analyzed with Annexin V-FITC kit (Nanjing Keygen Biotech, China) as previously described [18], [19]. Briefly, after the virus-infected cells had been treated with LiCl for 24 h, the apoptotic cells were determined by fluorescence microscopy (Leica, Germany) and the number of positive cells was calculated by selection of five view fields. The effect of LiCl on caspase-3 expression on virus-infected cells was analyzed using immunofluorescence (IF). The cells on 24-well plates were infected with TGEV for 1 h. Then the cells were incubated with 25 mM LiCl after three times washing with PBS. Then the cells were fixed with 4% formalin in PBS followed by quenching with 0.1 M glycine in PBS. The cells were incubated with specific rabbit anti-caspase-3 antibody (Beijing Biosynthesis, China) for 1 h followed incubation with fluoresceine isothiocyanate (FITC) conjugated goat anti-rabbit IgG (Zhongshan, China) for 1 h in the dark. Both antibodies were 1:100 diluted in 1% BSA. The nuclei were stained with propidium iodide (PI) for 15 min in the dark prior to microscopic observation.

In parallel, flow cytometry was used to further analyze cell apoptosis. Above-mentioned cell samples were trypsinized and then centrifuged at 2000 rpm for 5 min. The cells were re-suspended with 500** µ**l binding buffer at a concentration of 10^6^ cells/ml, after washing two times with PBS at 2000 rpm for 5 min. Then, 5** µ**l FITC-conjugated AnnexinV and 5** µ**l PI were added to the cells and incubated at room temperature for 15 min in the dark. The samples were analyzed within 1 h post-staining.

### Comparative analysis of anti-apoptotic effect between LiCl and pyrrolidine dithiocarbamate

The inhibitory effect of LiCl on cell apoptosis was comparatively analyzed with a caspase inhibitor, pyrrolidine dithiocarbamate (PDTC), a thiol reducing agent (Sigma, Beijing). Briefly, ST cells in 24-well plate were treated with 850** µ**M of H_2_O_2_ alone, or H_2_O_2_ (850** µ**M)+ PDTC (5** µ**M) or H_2_O_2_ (850** µ**M)+ LiCl (25 mM) at 37°C for 4 h. The mock treated cells were used as control. To analyze the inhibitory effect of PDTC and LiCl on TGEV induced cell apoptosis, ST cells in 24-well plate were infected with TGEV alone, TGEV+PDTC, or TGEV+LiCl at 37°C for 24 h. The mock-infected cells were used as control. The titer of TGEV applied was at an MOI of 0.3 and the final concentration of PDTC and LiCl was 5** µ**M and 25 mM, respectively. The mock-treated cells were used as control. All these cells were stained with Annexin V and PI and subjected to immunofluorescence analysis as above.

### Non-toxic concentration of LiCl

**Figure 1:**
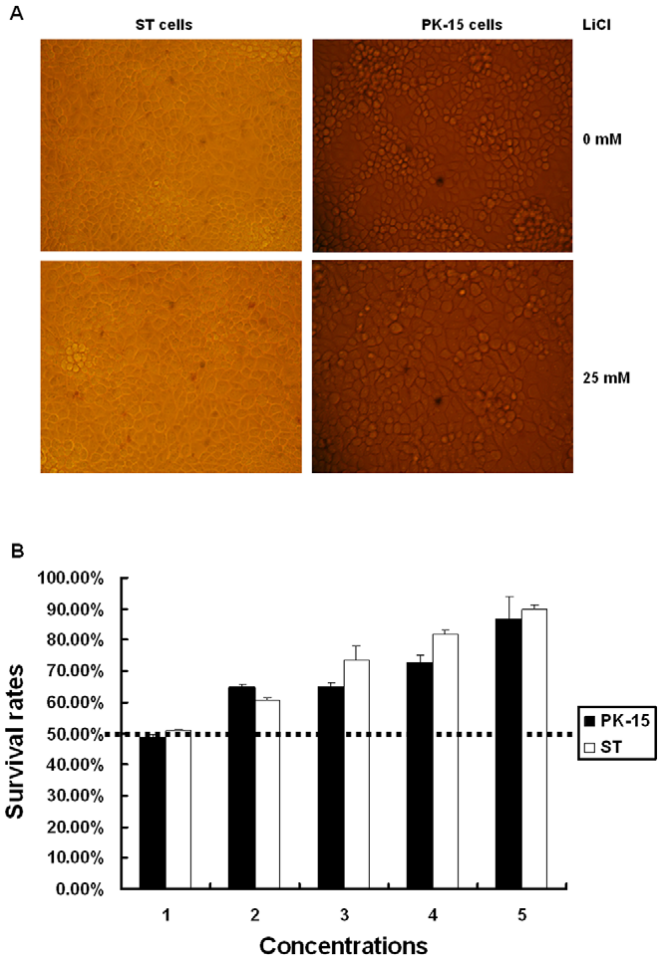
Cell viability analysis by trypan blue staining. The cell viability of two cell lines (ST and PK) was evaluated with trypan blue staining. Mock-treated cells and cells treated with the maximum non-toxic concentration of LiCl were subjected to trypan blue staining and a representative comparison is provided (A). The cell proliferation was analyzed by MTT colorimetric assays. The cell survival rates at different concentrations of drugs are given and 50% above cell survival rate (over broken line) is regarded as non-toxic concentration of LiCl. Concentrations 1 to 5 are 100, 50, 25, 20 and 10 mM for PK-15 cells respectively; 50, 30, 25, 20 and 15 mM for ST cells, respectively.

Staining with trypan blue showed that LiCl was non-toxic at concentrations up to 25 mM. The viability rates of the two cell lines analyzed, ST and PK cells, were above 95% and no morphological differences were observed between drug-treated and mock-treated cells at these concentrations (Figure 1). MTT assays that determine the activity of a mitochondrial enzyme and are commonly used to determine the proliferative activity of cells showed that proliferation of ST and PK cells was not affected by LiCl at concentrations below 25 mM (data not shown).

### Pretreatment of viruses or cells does not result in inhibition

To determine whether pretreatment of either cells or virus affects the viral infectivity, cells or the TGEV inoculum were treated with concentrations up to 25 mM of LiCl prior to infection. Subsequent infection of either LiCl-treated cells with untreated virus or untreated cells with LiCl-treated virus indicated that there is no inhibitory effect regardless whether infection was analyzed by plaque or MTT assays (data not shown).

### LiCl inhibits cell infection by TGEV

**Figure 2:**
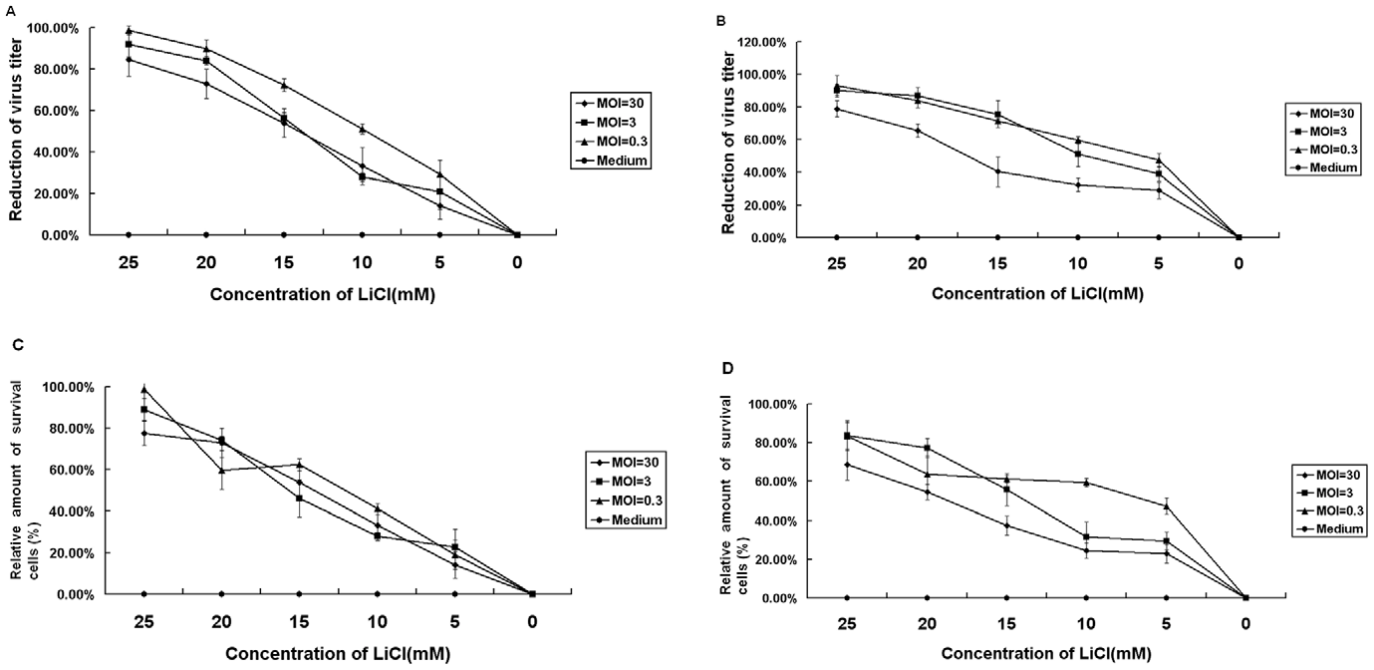
Protective effect of LiCl on TGEV infection of cultured cells. Increasing concentrations of LiCl were added to cells in 24-well plates infected with TGEV at different MOI at 1 hpi. At 48 hpi, the yield of virus in the supernatant was determined by plaque assays. The inhibition rates calculated from drug-treated ST and PK cells determined by plaque assays are shown in panels A and B, respectively. The effect of the infection was also determined by MTT assays. The relative amount of survival cells were calculated as described in M&M. The result obtained with ST and PK cells is shown in Panels C and D, respectively.

LiCl decreased cell infection by TGEV, when virus-infected cells were treated with LiCl. Plaque assays showed that some inhibition was observed at 5 mM LiCl. A concentration of 25 mM LiCl caused around 85% and 100% inhibition – depending on the virus dose in ST cell lines (Figure 2A) and between 80 and 90% inhibition in PK-15 cells (Figure 2B) infected by TGEV. The results were confirmed by MTT assays (Figures 2C and 2D). Although the inhibition of the virus at a higher MOI was slightly lower, LiCl had a similar antiviral activity at MOIs in the range from 0.3 to 30.

#### Time-of-addition effect of LiCl

**Figure 3:**
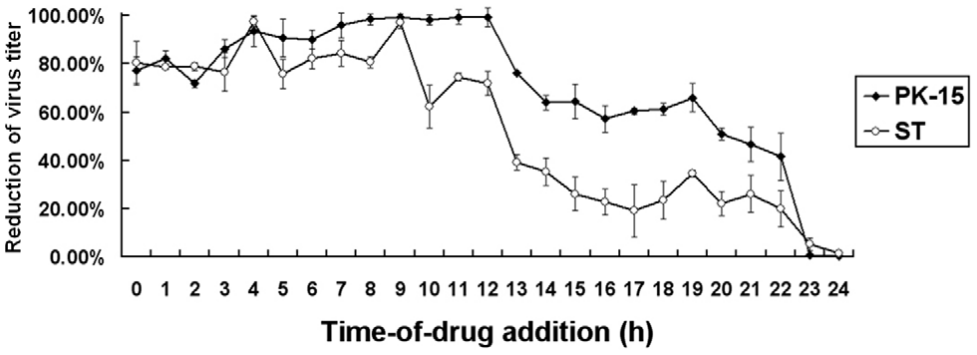
Time-of-addition effect of LiCl on TGEV replication. Cells in 96-well plates were infected with TGEV at an MOI of 30 followed by addition of 25 mM LiCl at the indicated time (hpi). The reduced virus titer was calculated using MTT assays by measuring OD_492_ value at 24 h post-infection. The experiment was performed in triplicate.

**Figure 4:**
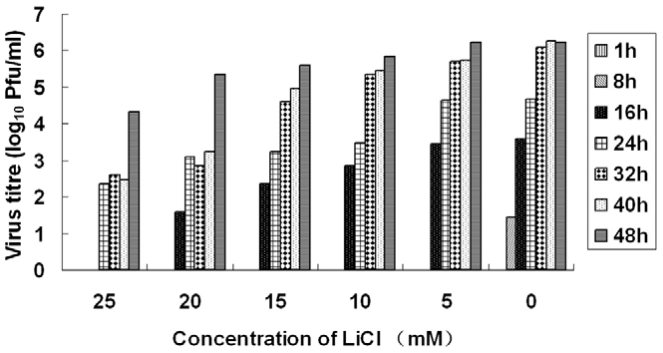
Virus production of virus-infected cells. ST cells were infected with TGEV at an MOI of 30 followed by incubation with serially diluted LiCl. The supernatant of the infected cells were collected at the different time points indicated and subjected to separate plaque assays.

The time-of-addition effect of LiCl on the infection cycle of TGEV was analyzed. The results showed that inhibition rates of 80% were observed when the drug was added within the first 12 h p.i. (Figure 3). As shown in Figure 4, the extent of inhibition by different concentrations of LiCl was the same whether virus production was assayed at 32 or 40 h.p.i. Even at 48 h.p.i, the inhibitory effect of the drug to TGEV-infected cells was still observed.

### RT-PCR and real-time RT-PCR analysis of the inhibitory effect of LiCl

**Figure 5:**
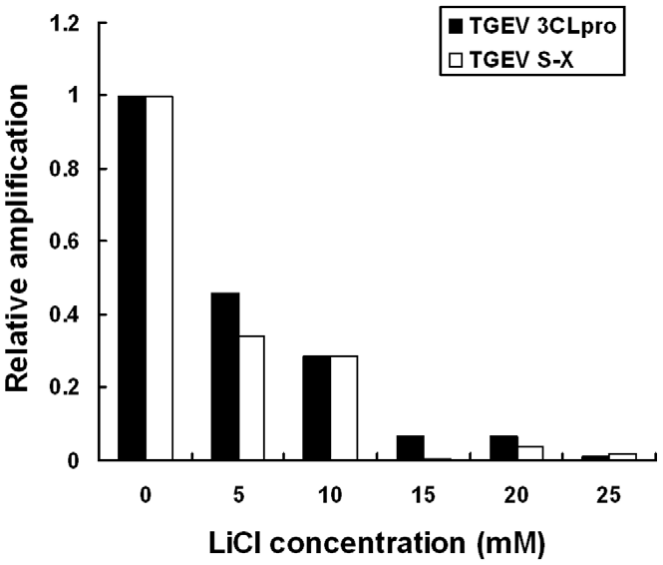
Quantitative measurement of TGEV 3CLpro and S-X gene. Viral RNA was extracted from cell lysates with or without LiCl treatment processed as described in M&M for real-time PCR. Reverse transcription was performed and the delta Ct value was measured at least in triplicate. The relative amplification of 3CLpro and TGEV-S-X in TGEV-infected cells was normalized to that of beta-actin and calculated using 2^−△△Ct^ method. Displayed results are average of at least three independent experiments.

The protective effect of LiCl on TGEV infection was confirmed on the RNA level by conventional RT-PCR. Amplification of S-AD (encoding the major antigenic sites A and D in the gene), S-X (partial TGEV S gene together with a partial 3a gene including the intervening sequence between both genes), and the gene for the nonstructural 3CLprotease (3CLpro). Analysis of the TGEV-RNAs indicated that the density of the product bands decreased with increasing concentrations of LiCl (data not shown). The effect of LiCl on the level of virus RNA was quantified by real-time RT-PCR. The result demonstrated the dose-dependent decrease of the amount of viral RNA synthesized in TGEV-infected cells. A major reduction (more than 50%) in the relative amplification of both TGEV S-X and 3CLpro genes was observed, when the cells were treated at a concentration of 5 to 25 mM of LiCl (Figure 5).

### Antiviral activities of LiCl against other RNA viruses

**Figure 6:**
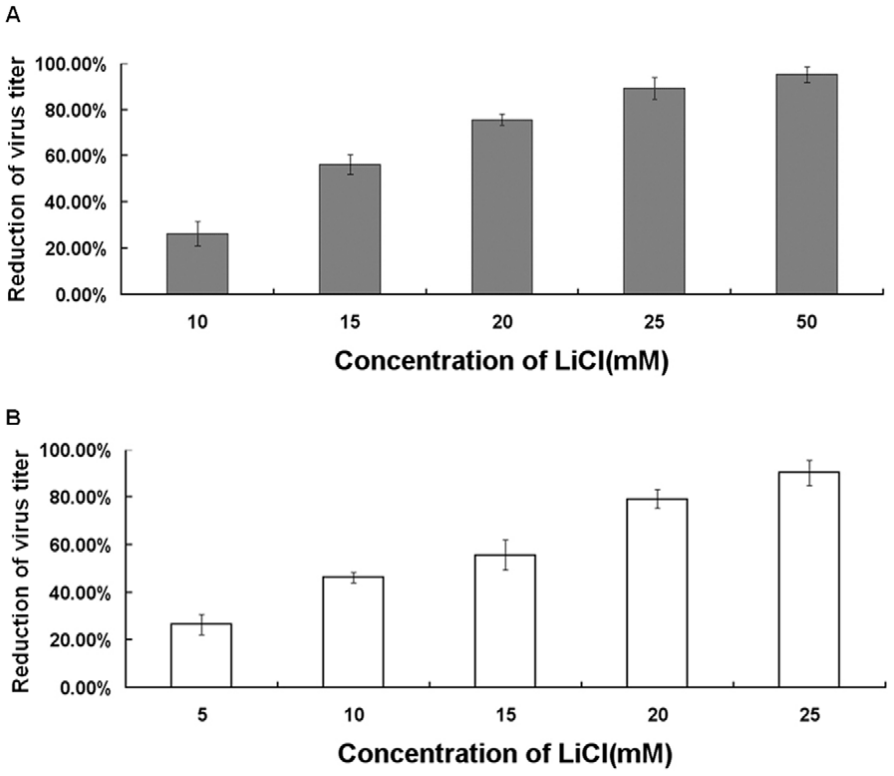
Antiviral activities of LiCl against other RNA viruses. The inhibitory effect of LiCl on two other RNA viruses, PEDV and BRV, in different cell lines was analyzed. The infectivity of PEDV and BRV was determined in Vero cells (A) and MA104 cells (B), respectively. The reduction of virus titer is indicated in the y axis.

To test whether LiCl also affects other RNA viruses, different concentrations of LiCl were analyzed for their effect on the infection by PEDV, another coronavirus, and by bovine rotavirus (BRV). The former virus was analyzed in Vero cells (Figure 6A), the latter virus in MA104 cells Figure 6B). The maximum non-toxic concentrations of LiCl in these cells are 50 mM and 25 mM, respectively (13, 27, unpublished data). At these concentrations, the infection was inhibited by more than 95% (Figure 6).

### LiCl inhibits TGEV-induced cell apoptosis

**Figure 7:**
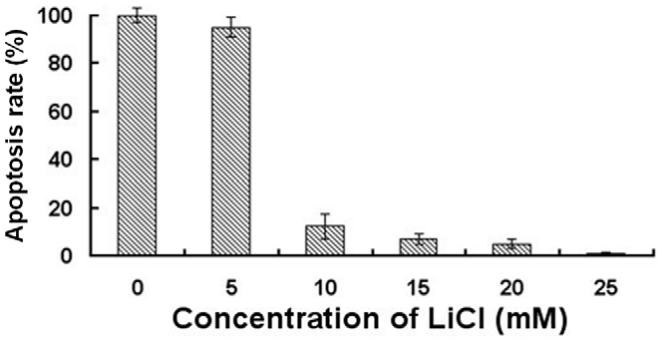
Inhibitory effect of LiCl on cell apoptosis analyzed by immunofluorescence. The cell apoptosis was analyzed after virus-infected ST cells were treated with LiCl at different concentrations. Mock-treated cells were used as control. The cells were stained with FITC-conjugated Annexin V and the fluorescence signals were detected under fluorescence microscope. The calculated apoptosis rate is provided.

**Figure 8:**
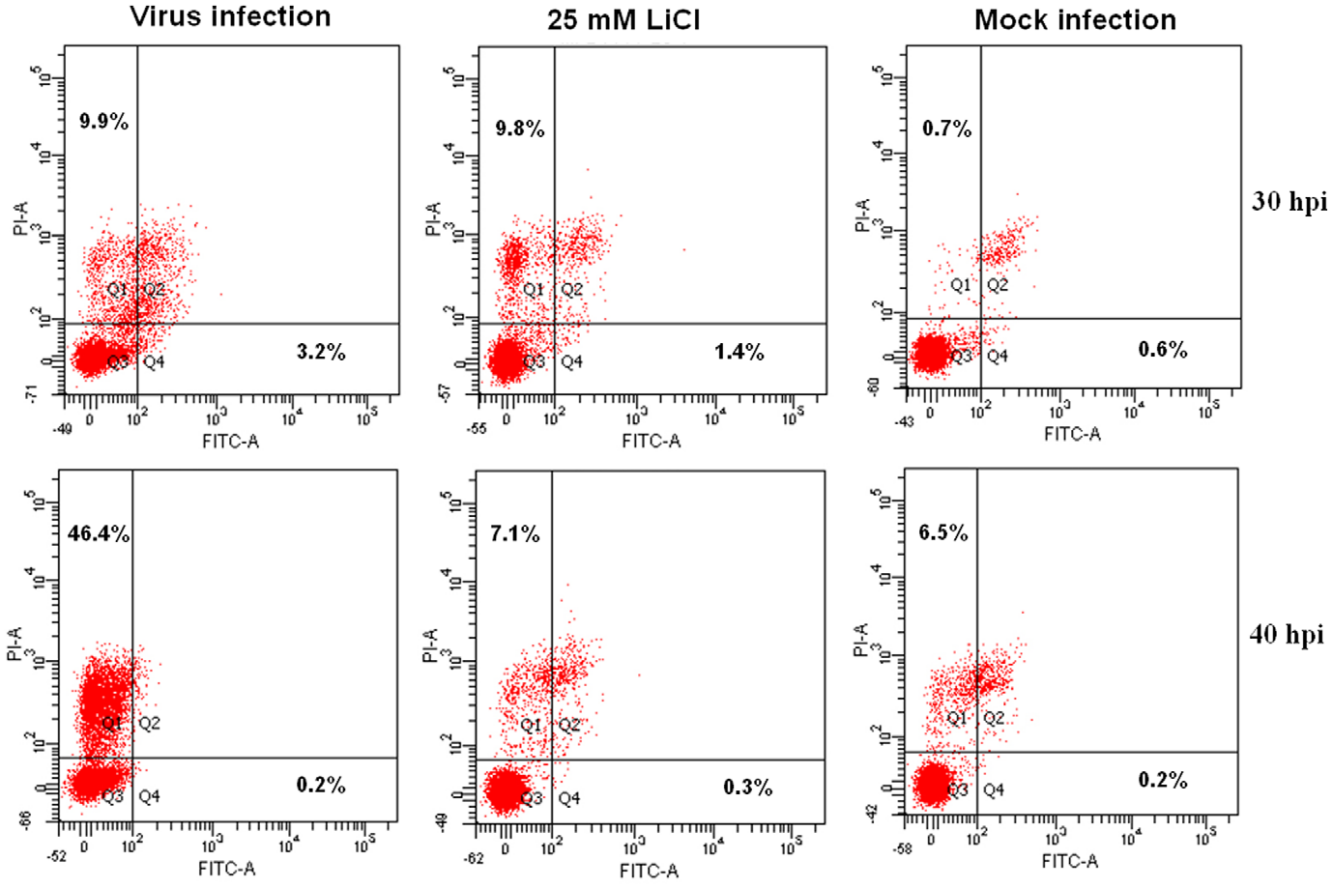
Cell apoptosis analysis by flow cytometry. The virus-infected cells were stained with Annexin V-FITC and PI, after they had been treated with 25 mM LiCl. The cell apoptosis was analyzed by flow cytometry at either 30 or 40 h.p.i. The early and late apoptosis was quantified and indicated in Q4 and Q1 gates, respectively. The apoptosis rates of cells are indicated.

Apoptosis is a characteristic feature of TGEV-infected cells. Therefore, we were interested whether LiCl is able to prevent the apoptotic effect of TGEV. Apoptotic cells were visualized by staining for annexin. As shown in Figure 7, counting the apoptotic cells at 30 hpi. revealed that LiCl decreases TGEV-induced apoptosis in a dose-dependent manner. Further quantification of apoptotic cells was performed with flow cytometry. The results showed that at 30 hpi, the early apoptosis of TGEV-infected cells were significantly decreased after treatment with 25 mM LiCl. At 40 hpi, most cells were at a stage of late apoptosis, which was also inhibited by drug treatment (Figure 8).

**Figure 9:**
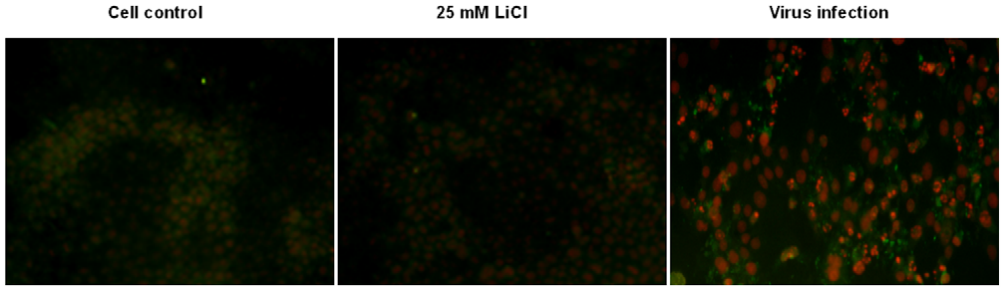
Expression of caspase-3 detected by immunofluorescence. The expression of caspase-3 on cells was analyzed using immunofluorescence. ST cells on 24-well plates were infected with TGEV for 1 h. Then the cells were treated with LiCl for 40 h followed by incubation with anti-caspase-3 antibody and FITC-conjugated secondary antibody. The nuclei were stained with propidium iodide prior to microscopic observation.

**Figure 10:**
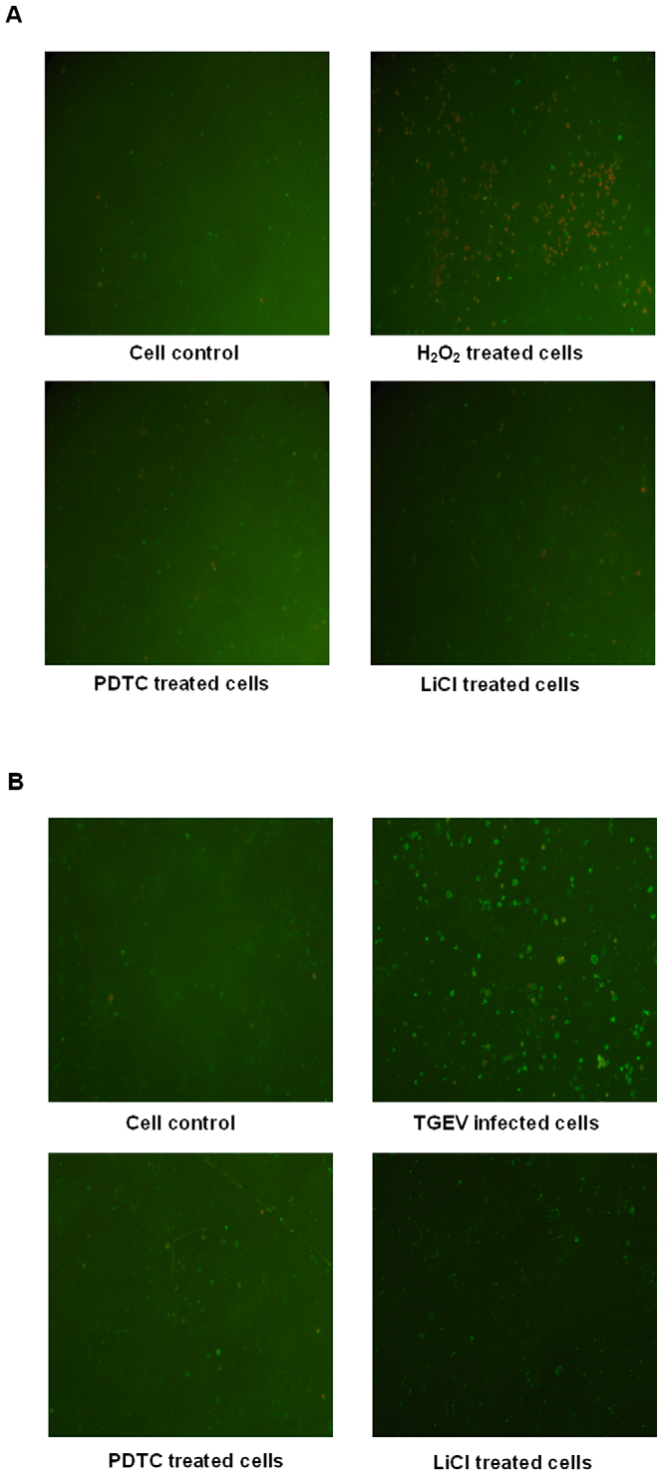
Immunofluorescence assays of the inhibitory effect of LiCl and PDTC on cell apoptosis. The cell apoptosis was induced by H_2_O_2_ and the anti-apoptosis effect of LiCl was compared with a caspase inhibitor, pyrrolidine dithiocarbamate (PDTC). ST cells in 24-well plate were mock treated (cell control) or treated with 850** µ**M of H_2_O_2_ alone (H_2_O_2_ treated cells), or H_2_O_2_ (850** µ**M)+ PDTC (5** µ**M) (PDTC treated cells) or H_2_O_2_ (850** µ**M)+ LiCl (25 mM) (LiCl treated cells) at 37°C for 4 h followed by staining with Annexin V and PI and subjected to immunofluorescence analysis (A). The inhibitory effect of both agents on TGEV induced cell apoptosis was evaluated. ST cells in 24-well plate were mock infected (Cell control) or infected with TGEV alone (TGEV infected cells), TGEV+PDTC (5** µ**M) (PDTC treated cells), TGEV+LiCl (25 mM) (LiCl treated cells) at 37°C for 24 h. The titer of TGEV applied was at an MOI of 0.3 followed by Annexin V and PI staining and subjected to immunofluorescence analysis (B). Green and red show the early and late apoptotic cells, respectively.

Caspase-3 is an enzyme involved in the apoptosis of cells. We were interested to know, whether the antiapoptotic effect of LiCl may be explained by inhibition of caspase-3. As shown in Figure 9 by immunofluorescence microscopy, the expression of caspase-3 was induced in TGEV-infected cells, but drastically reduced in cells treated with LiCl. Like PDTC, LiCl was a potent apoptosis inhibitor in terms of the inhibitory effect on cell apoptosis induced by H_2_O_2_ (Figure 10A). Both agents also decreased the number of apoptotic cells induced by TGEV effectively (Figure 10B).

## Discussion

TGEV is an important pathogen in pigs, causing high mortality in piglets. Generally, the SIgA in colostrum of sows vaccinated by virulent or attenuated vaccine can provide effective protection to suckling piglets; however, the potential dissemination and prevalence of infectious agent in piglets remains. In addition to enhancement of vaccine safety and efficacy, development of potential antivirals is a complementary approach in the future strategy for preventing TGEV infection. There are very limited reports concerning anti-TGEV drugs and lack of effective therapeutical treatment highlights the importance of identifying appropriate antivirals. Recently, Yang et al. (2007) used biochemical and virological methods to screen small-molecule compounds that inhibit TGEV replication. The authors found a series of benzothiazolium compounds that had inhibitory activity against TGEV 3CLpro, one of the main proteases for the replicase polyproteins in coronaviruses and these drugs exerted anti-TGEV activities in terms of viral protein synthesis and RNA replication in TGEV-infected ST cells [25]. More recently, LiCl was found to be effective in preventing infection by IBV, an avian coronavirus, *in vitro* [17], [18]. The findings regarding the inhibitory effect of LiCl on IBV are novel, because an inhibitory effect had been reported previously only on several DNA viruses.

To see whether coronaviruses from different species have a general susceptibility to LiCl treatment, we performed an evaluation of the antiviral effect of LiCl on TGEV, a porcine coronavirus. First, we used two approaches to analyze TGEV infection in two cell lines (porcine kidney cell line, PK-15 and swine testis cell line, ST). The inhibitory effect of LiCl was evident both when the virus was titrated by plaque assay and when the viability of the cells was determined by the MTT assay quantifying mitochondrial dehydrogenase activities. In addition to TGEV strain PUR46-MAD, a virulent TGEV isolate HBY was tested for its sensitivity to LiCl and was found also to be affected by this inhibitor (data not shown). At the same time, infection of different cell lines by PEDV, another porcine coronavirus and bovine rotavirus (PRV), a double-stranded RNA virus, were inhibited by LiCl effectively. These data show that inhibition by LiCl may be a general feature of some virus families, e.g. coronaviruses. In addition, we found that LiCl did not directly interact with or inactivate TGEV particles and was ineffective when pre-incubated with host cells. In contrast, it inhibited plaque formation of virus-infected cells. The results are consistent with the inhibitory effect of LiCl on IBV. We therefore confirm our hypothesis that the sensitivity of coronavirus to LiCl is a general feature of this virus family.

It has been suggested that LiCl may act as an inhibitor of the DNA virus herpes simplex virus [15]. Viral proteases play key roles in virus replication and have been identified as potential targets in the search for antiviral agents, e.g. for treatment of human immunodeficiency virus or hepatitis C virus infections [25]–[28]. In our study, the conventional RT-PCR targeting the 3CLpro and a 5′ leader sequence of 3a gene in conjunction with the partial TGEV S gene indicated that the effect of LiCl could be on RNA transcription and replication. For IBV and TGEV coronaviruses, it is also possible that LiCl inhibits RNA-dependent RNA polymerases, which are characteristic of positive and negative-stranded RNA viruses [17]. To further address the action of LiCl on TGEV replication, the plaque formation and virus production were quantified using time-of-addition assays during the infection cycle of TGEV. Our results showed that LiCl inhibited TGEV infection effectively when added as late as 12 h after the virus inoculation, indicating that the inhibition of LiCl occurred during the whole viral replication after infection. In our study, we used different TGEV titers for cell infection and the inhibition of TGEV by LiCl *in vitro* was titer-independent, which implies that the drug also inhibits virus spread from cell to cell. The impact of LiCl on TGEV load evaluated by real-time PCR further confirmed its inhibition activity against TGEV.

Recently, the antiviral activity of quercetin 7-rhamnoside against PEDV, another porcine coronavirus, has been analyzed. Interestingly, the effectiveness of Q7R against TGEV and PRCV (a respiratory variant of TGEV) was lower when compared to PEDV [29]. This finding might suggest that some coronaviruses differ in their susceptibility to the same drug. In our study, we show that coronaviruses TGEV and PEDV are equally sensitive to LiCl treatment; BRV, another RNA virus, showed a similar sensitivity to the inhibitor. Our findings suggest a consistent sensitivity of coronaviruses and some other RNA viruses to LiCl. TGEV is the first coronavirus documented to trigger apoptosis in infected cells [30]. Subsequently, infectious bronchitis virus (IBV) [31], murine hepatitis virus (MHV) [32], severe acute respiratory syndrome-associated coronavirus (SARS-CoV) [33], and equine coronavirus [34] were found to have similar apoptotic effects. In our study, we confirmed that TGEV infection induced the appearance of apoptotic cells as shown by immmunofluorescence analysis. The flow cytometry results showed that most apoptotic cells were at a stage of late apoptosis at 40 h post-infection; however, both early and late apoptosis of TGEV-infected cells was inhibited by LiCl efficiently. Caspase-3, a member of the aspartate-specific cysteinyl proteases or caspases has been identified as being a key mediator of apoptosis of mammalian cells [35]. In this study, the expression of caspase-3 was inhibited after addition of LiCl to TGEV-infected cells. PDTC, a known caspase inhibitor, was able to inhibit TGEV induced apoptosis [30]. Interestingly, we found that LiCl also had a potent inhibitory effect on TGEV-induced cell apoptosis. Apoptosis-associated caspase activation of TGEV and SARS-CoV has been reported [30], [33]. We therefore anticipate that LiCl may have protective effects on SARS-CoV induced infection and apoptosis. Taken together, the data indicate that LiCl may inhibit TGEV infection via exerting an anti-apoptotic effect mediated by caspase 3-related pathways.
